# Supplementary material for: Radiosensitivity of Cancer Cells Is Regulated by Translationally Controlled Tumor Protein
Source: Cancers (Basel). 2019 Mar 19;11(3):386. doi: 10.3390/cancers11030386 (PMC6468585; doi:10.3390/cancers11030386)
Supplement: Supplementary file 1 [file cancers-11-00386-s001.pdf]

Supplementary Materials: Radiosensitivity of Cancer Cells Is Regulated by Translationally Controlled Tumor Protein

Jiwon Jung, Ji-Sun Lee, Yun-Sil Lee and Kyunglim Lee

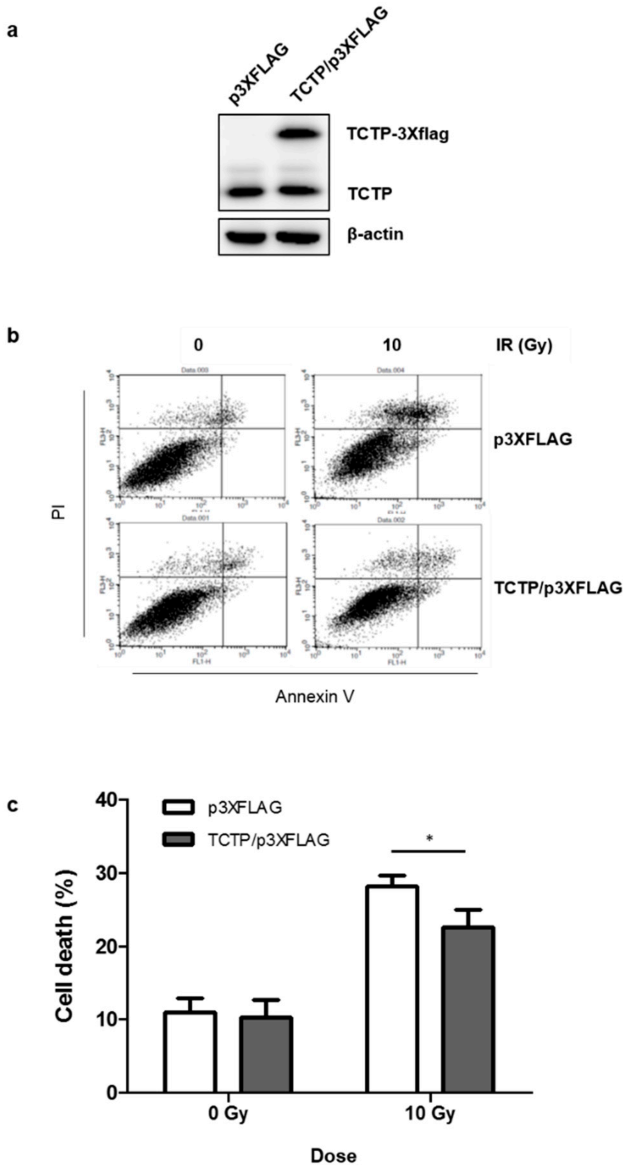

Figure S1. TCTP overexpression increases radioresistance in H460 cells. (**a**) H460 cells were transfected with TCTP-3Xflag, and the overexpression of TCTP-3Xflag was confirmed using western blotting. TCTP-3Xflag overexpressed H460 cells were treated with γ-radiation of 10 Gy, and cell death was analyzed after 48 h. (**b**) The representative images of PI-Annexin V double staining examined in TCTP overexpressed H460 cells and (**c**) the graph of cell death is shown. Values are means ± SEM. * $p < 0.05$ ($n = 4$).

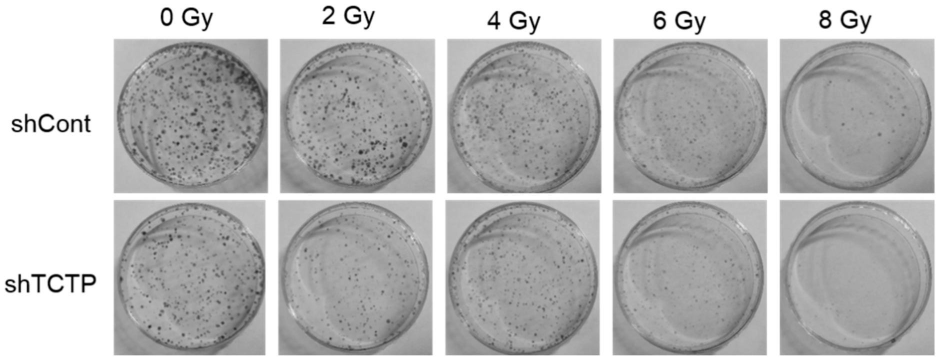

Figure S2. Representative images of clonogenic formation assay of control and TCTP knock downed A549 cells.

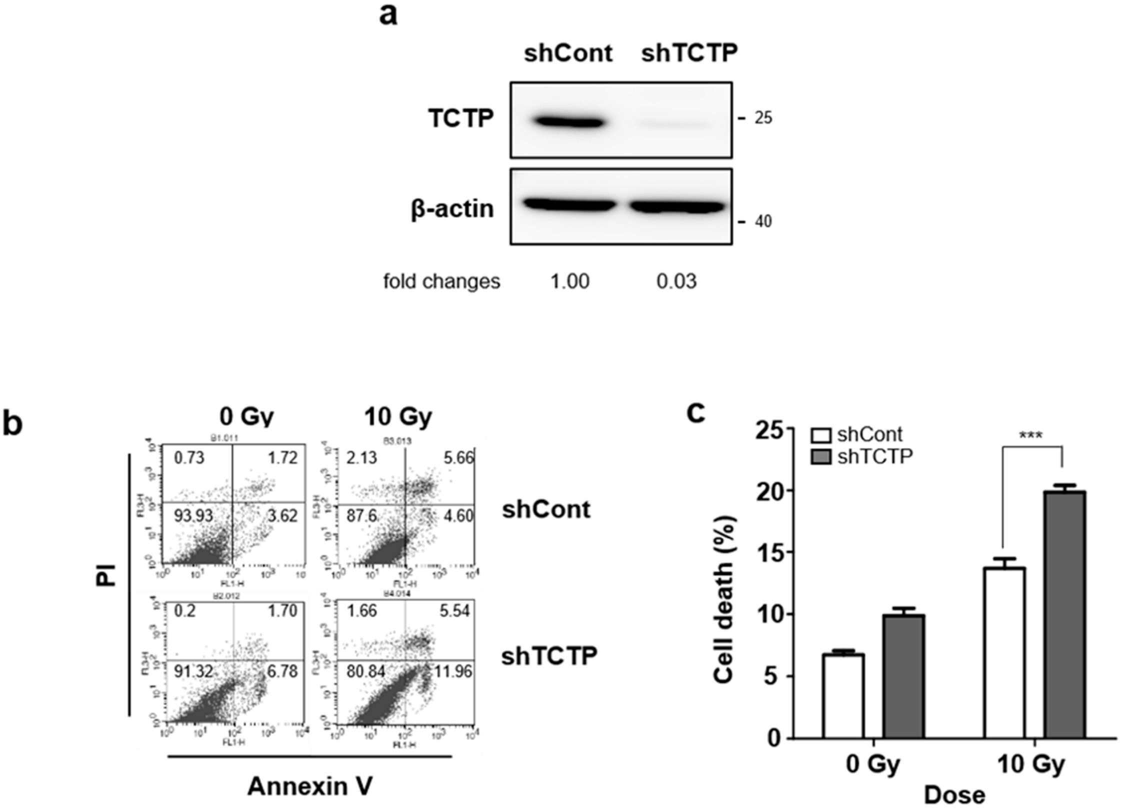

Figure S3. Stable knockdown of TCTP decreases radioresistance in A549 cells. (**a**) Stable cells expressing shRNA for TCTP was generated by pLKO.1 lentiviral system and knockdown efficiency was confirmed by Western blotting. Each cell was treated with γ-radiation of 10 Gy, and cell death was analyzed after 48 h. (**b**) The representative images of PI-Annexin V double staining and (**c**) the graph of cell death is shown (n = 3). Values are means ± SEM. *** $p < 0.001$ by two-way analysis of variance.

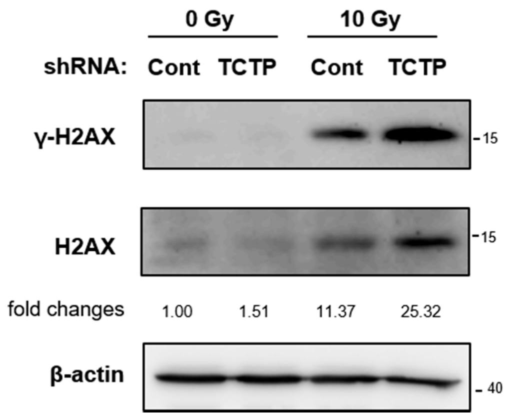

Figure S4. TCTP knockdown enhances radiation-induced phosphorylation of H2AX. Cells that express shRNA (Cont and TCTP) stably were exposed to γ-radiation and γ-H2AX and H2AX expression levels were measured using Western blot analysis.

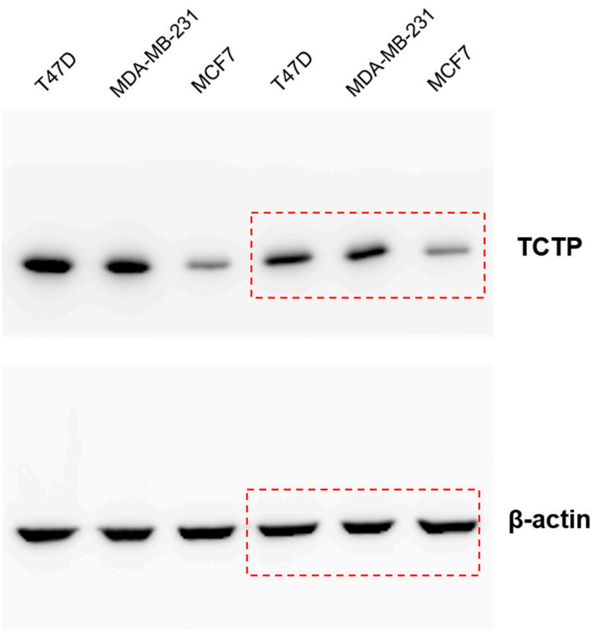

Figure S5. Whole membrane images for Figure 1a.

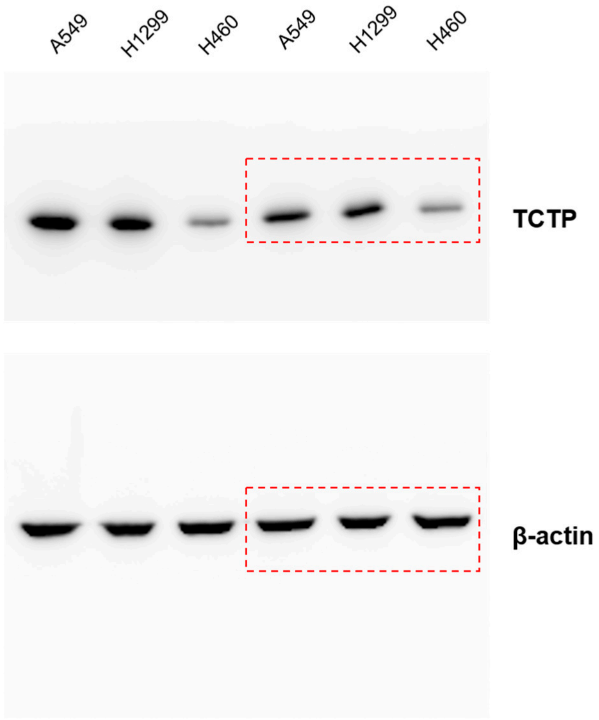

Figure S6. Whole membrane images for Figure 2a.

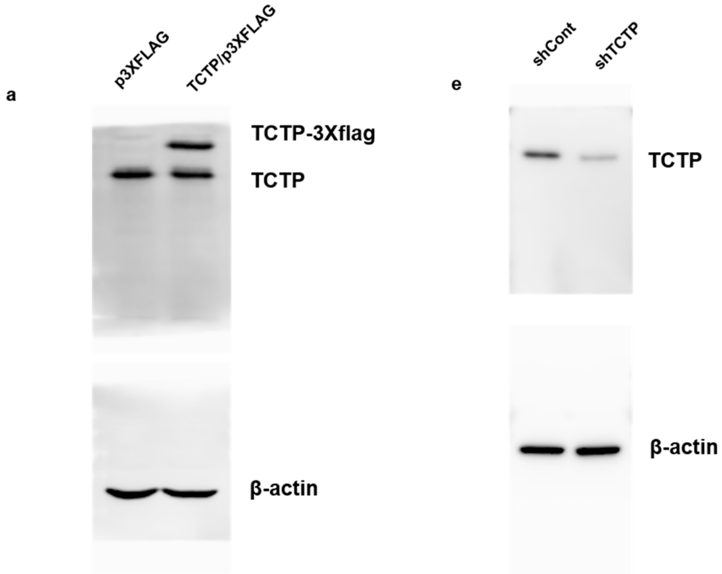

Figure S7 Whole membrane images for Figure 3a and 3e

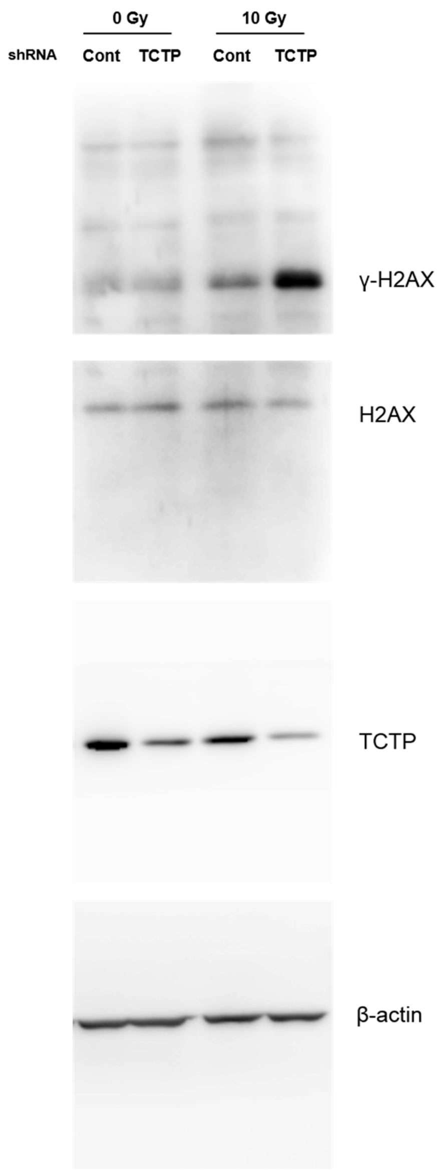

Figure S8. Whole membrane images for Figure 4a.

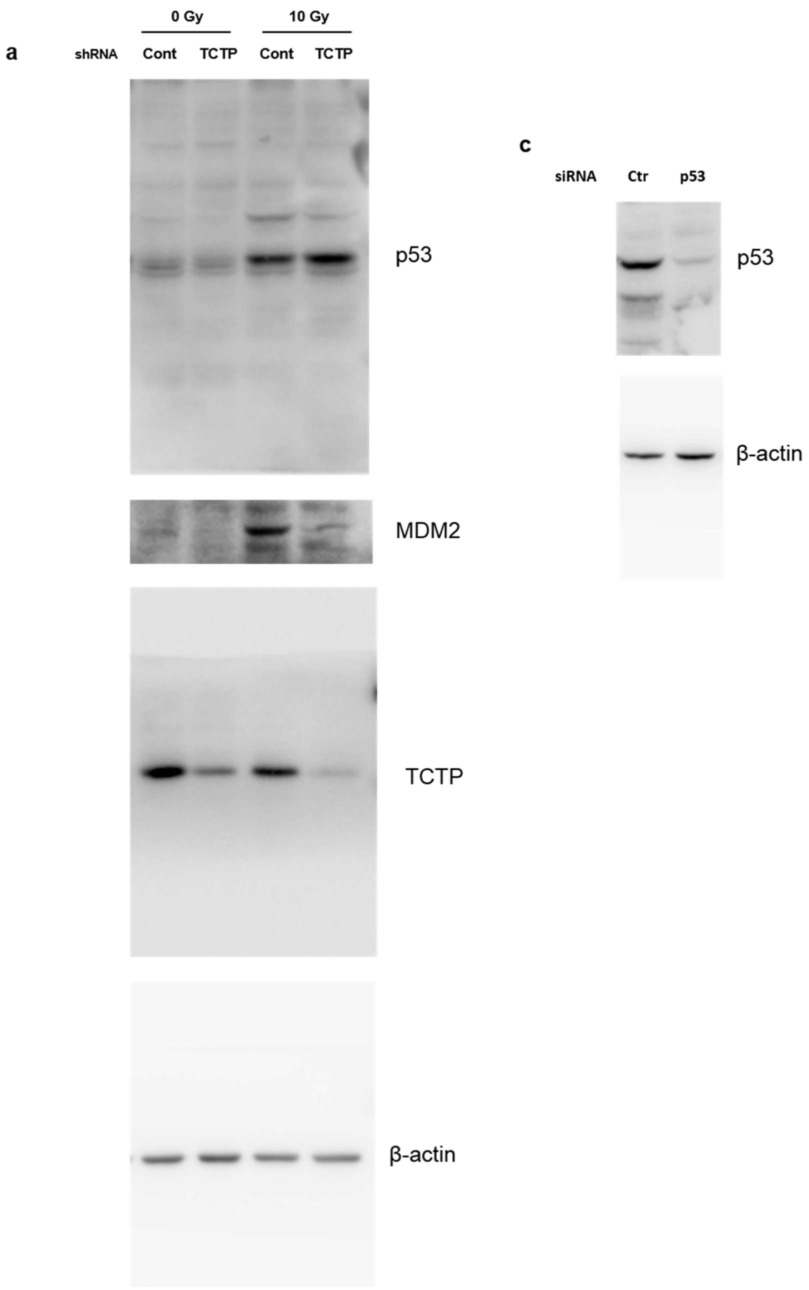

Figure S9. Whole membrane images for Figure 5a and 5c.

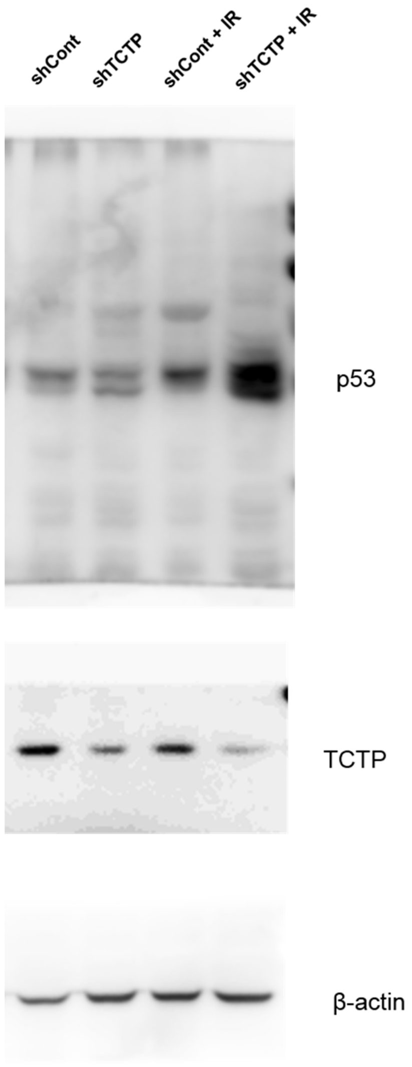

Figure S10. Whole membrane images for Figure 6c.

© 2019 by the authors. Licensee MDPI, Basel, Switzerland. This article is an open access article distributed under the terms and conditions of the Creative Commons Attribution (CC BY) license (http://creativecommons.org/licenses/by/4.0/).
